# Joint spatial modelling of disease risk using multiple sources: an application on HIV prevalence from antenatal sentinel and demographic and health surveys in Namibia

**DOI:** 10.1186/s41256-017-0041-z

**Published:** 2017-08-01

**Authors:** D. Ntirampeba, I. Neema, L. N. Kazembe

**Affiliations:** 1 Department of Mathematics and Statistics, Namibia University of Science and Technology, Windhoek, 2064 Namibia; 2 Namibia Statistics Agency (NSA), Windhoek, 2064 Namibia; 3 Department of Statistics and Population Studies, University of Namibia, P/Bag 13301 Pionerspark, Windhoek, 2064 Namibia

**Keywords:** HIV prevalence, Joint (bivariate) analysis, Spatial analysis, Namibia

## Abstract

**Background:**

In disease mapping field, researchers often encounter data from multiple sources. Such data are fraught with challenges such as lack of a representative sample, often incomplete and most of which may have measurement errors, and may be spatially and temporally misaligned. This paper presents a joint model in the effort to deal with the sampling bias and misalignment.

**Methods:**

A joint (bivariate) spatial model was applied to estimate HIV prevalence using two sources: 2014 National HIV Sentinel survey (NHSS) among pregnant women aged 15–49 years attending antenatal care (ANC) and the 2013 Namibia Demographic and Health Surveys (NDHS).

**Results:**

Findings revealed that health districts and constituencies in the northern part of Namibia were found to be highly associated with HIV infection. Also, the study showed that place of residence, gender, gravida, marital status, number of kids dead, wealth index, education, and condom use were significantly associated with HIV infection in Namibia.

**Conclusion:**

This study had shown determinants of HIV infection in Namibia and had revealed areas at high risk through HIV prevalence mapping. Moreover, a joint modelling approach was used in order to deal with spatially misaligned data. Finally, it was shown that prediction of HIV prevalence using the NDHS data source can be enhanced by jointly modelling other HIV data such as NHSS data. These findings would help Namibia to tailor national intervention strategies for specific regions and groups of population.

## Background

Although a downwards change in the trajectory of AIDS epidemic has been achieved worldwide [1], by the end of 2014, 36.9 million people were estimated to live with HIV [2], of which about 70% (25.8 million) are found in sub-Sahara Africa. In 2014, it was estimated that the global total of 2 million of people were newly infected with HIV, a large portion (1.4 million) of which is said to be in sub-Sahara Africa [2].

Namibia is one country where the HIV prevalence is high [3]. In 2014, the number of people living with HIV among adults and children was estimated to be around 26,000, of which 11,000 were newly infected [3]. The National HIV Sentinel survey (NHSS) and Namibia Demographic and Health Survey (NDHS) are the commonly used tools to monitor the prevalence HIV trend in the country. Indeed, the analyses of data resulting from these surveys are vital in generating strategic information for evaluating the effectiveness of programs and policies and enabling to improve and redesign programmes. However, each one of the two data sources has its own weaknesses that may lead to inaccurate estimation of HIV prevalence. For the former, limitations such as accessibility of ANC sites and exclusion of some categories of the population (e.g. men and non-pregnant women) are well documented [4]. The latter suffers most of times from a significant non-response drawback [4].

In the face of these limitations, a joint analysis of data from different sources has been proven to be useful [5]. It avoids multiple testing on same data, helps dealing with identifiability in random effect parameters estimation, and increases precision and efficiency of parameter estimates. Further, the multivariate analysis technique can help to capture disease specific covariates and as well as to carry pairwise and cross-covariances inferences between different sources [5]. Different approaches of multivariate techniques that include the multivariate normal distribution, iterative generalised least squares (IGLS) method, multivariate conditional autoregressive (MCAR) modelling, and the shared-component modelling are commonly used in mapping of multiple diseases. Although multivariate normal and IGLS methods allow modelling different sources simultaneously, these two methods underestimate the variation associated with sources [5]. In spatial disease mapping, one way to account for within and/or between areal associations is to employ MCAR modelling approach [5]. But due to high parameterisation, the computation and interpretation of parameters becomes cumbersome. Recent applications of MCAR modelling approaches include [6, 7]. Recently, shared- component model pioneered by Knorr-Held and Best [8] had been extensively used in joint analysis of multiple health outcomes (e.g. [4, 5, 8–11]). This model splits the disease profile into two components, namely the disease-specific component representing spatially varying factors, and the shared component which is a proxy of unobserved spatially varying factors that are common to both or all diseases [8]. Bellier et al. [12] had jointly analysed multiple data sources by including an observability parameter. Guo and Carlin [13] had used a full Bayesian approach to link longitudinal and survival data. Other recent examples of jointly modelling multiple data sources include [14–18].

Even though there is a rich literature on analyses of determinants of HIV and its geographical spread, most of the analyses used were based on univariate methods for different data sources. One notable study by Manda et al. [4] had used a shared-component modelling approach to jointly analyse data from NDHS and ANC surveys. For the two sources, district level HIV prevalence rates were used and also two contextual covariates were considered as determinants of HIV. In other words, in their study, the data were first aggregated at district level and then a spatial bivariate modelling approach was applied on aggregated rates. In this situation, a misalignment in data sources is avoided. However, this has some limitations as, for instance, many covariates available from ANC or NDHS would not be used in the joint analysis. One way to include most of ANC and/or NDHS covariates would be first to compute averages at district level. Alternatively, a model that allows different neighbourhood structures may be useful as it would permit to model data available at different block levels.

A primary objective of this study is to develop a joint spatial model for NHSS and NDHS data, which enables the estimation at any location of the constituency or district level while dealing with misalignment in data.

## Methods

### Data

Two data sets were used in this study, namely, the 2013 Namibia Demographic and Health Survey (NDHS) data and the 2014 National HIV Sentinel survey (NHSS) data from women aged 15–49 attending antenatal care clinics (ANC). Table 1 provides a list of all variables used in this study, as identified through the literature [4, 6].

**Table 1:** Summary of variables used in the study by source

	NDHS	NHSS
Variables	1. HIV status	1. HIV status
	2. Place of residence	2. Age of the respondent
	3. Gender	3. Number of children born by a mother (gravidity)
	4. Age of the respondent	
	5. Head of household	
	6. Marital status	
	7. Number of kids dead	
	8. Education	
	9. Wealth	
	10. Stayed away of home	
	11. Sexual activity (in last 4 months)	
	12. Age at first sex	
	13. Condom use	
	14. Had STI in last 12 month	

### NDHS data

The sampling methodology for the 2013 Namibia Demographic and Health Survey was a two stage stratified cluster survey design. In the first stage, 554 enumeration areas (EAs) were selected using probability proportional to size of EA, with stratification into rural and urban areas. In the second stage, 20 households were selected from each EA using equal probability systematic sampling approach. One of the key objectives of this survey was the collection of data on knowledge and prevalence of HIV/AIDS and other diseases such as diabetes, cardiovascular disease, cancer, and chronic respiratory disease [19]. To achieve this objective, the survey included three questionnaires (Household questionnaire, woman’s questionnaire, and the man’s questionnaire) that addressed questions on household characteristics and assessed women’s and men’s knowledge of HIV. A total 9176 women and 3950 men formed part of 2013 NDHS interviews. Further, the survey included HIV testing amongst women and men aged between 15 and 64 years selected throughout the country. Details on the survey methodologies used in collecting data can be obtained from the 2013 NDHS report [19]. The variables resulting from this survey were grouped into four categories, namely, demographic, social, biological, and behavioural. The sample for the survey is thought to be a representative of the general population and also provides a vast range of population and demographic characteristics useful in the study of HIV prevalence and its related determinants.

### NHSS data

Since 1992, every second year, a National HIV Sentinel survey (NHSS) has been conducted by Ministry of Health and Social Services (MoHSS) in order to determine HIV prevalence among pregnant women aged 15–49 years attending antenatal care (ANC) clinics at public health facilities in Namibia. Since its inception, the NHSS has expanded from 8 sites to 35 district sites supplemented by 98 satellite facilities. The main objectives of NHSS is to obtain reliable data that can be used to assess the national prevalence of HIV among pregnant women in age group of 15–49 years; to identify socio-demographic covariates associated with high prevalence; and to fast-track the estimation of the spatial and temporal prevalence trends. Sampling techniques, sample size and data collection methods were based on the World Health Organization (WHO) guidelines for conducting HIV surveys among pregnant women and other groups [3]. For more details, the reader can refer to the surveillance reports of National HIV sentinel survey [3]. In this study, the 2014 NHSS, which was conducted from 10 March to 30 September 2014, was used. In total, of 7920 women enrolled in the 2014 NHSS, the majority of them were multi-gravida. In the data, the following variables were collected: age, gravidity, district, and HIV status. Though not many covariates are provided by NHSS, it brings an important contribution in terms of HIV prevalence to this study as not many non-response cases are experienced in comparison to the NDHS. Table 1 gives a summary of variables obtained from both NDHS and NHSS used in this study.

## Statistical models

### Univariate modelling of data

The univariate modelling approach was achieved by fitting a separate model for each data source as follows. Let y_ij_ be a binary indicator of HIV incidence at location i ( s_i_) from dataset j such that y_ij_ is one if a disease incident is observed at location i for dataset j and zero elsewhere. In here, the location i could be a health district facility in a health district (for NHSS data source) or a location in a constituency (for NDHS data). Then y_ij_ ~ Bernouilli(p_ij_), where p_ij_ is the probability of a recorded incident at location i from dataset j. Thus, the independent model fitted to dataset ( j=1, 2) is given by;

1 $$ logit\left({p}_{i j}\right)={\beta}_{0 j}+{\sum}_k^r{\beta}_k{x}_{i j k}+{f}_j\left({g}_i\right)+{z}_j\left({s}_i\right), $$

with β_0j_ represents the model intercept, x_ijk_ is the k^th^ linear covariate of dataset j in a given health district facility i or constituency i, f_j_(.) is a function of a non-linear covariate, g_i_ is a vector of ages, and z_j_(s_i_) is Gaussian random field. Eq. (1) can be split into two separate (univariate) models as follows. At the first stage of Bayesian hierarchy,

2 $$ logit\left({p}_{i1}\right)={\beta}_{01}+{\sum}_k^r{\beta}_k{x}_{i1 k}+{f}_1\left({g}_i\right)+{z}_1\left({s}_i\right), $$

3 $$ logit\left({p}_{i2}\right)={\beta}_{02}+{\sum}_k^r{\beta}_k{x}_{i2 k}+{f}_2\left({g}_i\right)+{z}_2\left({s}_i\right), $$

For the Gaussian random field, it was assumed a multivariate Gaussian distributionz (*s*) ~ *N*(0, Σ), where Σ is the covariance matrix. The elements of the covariance matrix Σ are specified as a function of the marginal variance of the process *σ*_*z*_ and the Mat $\dot{e}$ rn correlation function *Cor*_*M*_ as follows:

Σ_*ij*_ = *σ*_*z*_ *Cor*_*M*_(*z*(*s*_*i*_), *z*(*s*_*j*_)).

The Matern correlation function is given by:

4 $$ {Cor}_M\left( z\left({s}_i\right), z\left({s}_j\right)\right)=\frac{2^{1-\nu}}{\Gamma \left(\nu \right)}{\left( k\left\Vert {s}_i-\right.\left.{s}_j\right\Vert \right)}^{\nu}{K}_{\nu}\left( k\left\Vert {s}_i-\right.\left.{s}_j\right\Vert \right), $$

where ‖.‖ denotes the Euclidean distance, *K*_*ν*_(.) is the modified Bessel function of second order, *k* and *ν* are scale parameter and smoothness parameter, respectively.

At the second stage of Bayesian hierarchy, inverse Gamma prior distributions were assigned to *k*, *ν*, and *σ*_*z*_. For fixed effect parameters *β*, the study assumed weakly informative Gaussian priors $\beta \sim N\left(0,{\tau}_{\beta}^{-1} I\right)$ with small precision *τ*_*β*_ on identity matrix. In order to deal with non-linearity effects of continuous covariates (ages), Δ*g*_*i*_ was assumed to follow a first order random walk process (i.e. Δ*g*_*i*_∣Δ*g*_*i* − 1_ ~ *N*(Δ*g*_*i* − 1_, *σ* ^2^)). Alternatively, a semi parametric model that uses the penalised regression spline approach may be used and details of the penalised regression approach can be found elsewhere [6, 20].

### Joint modelling of HIV prevalence from NDHS and NHSS data sources

In the joint (bivariate) setting, the HIV prevalence from NDHS data source and the HIV prevalence from NHSS data source were modelled jointly instead of fitting separate model for each data source. In this study, a bivariate modelling approach was applied using the spatial shared component model that incorporated information from NHSS source that might be common to NDHS data source in order to improve the estimation of HIV prevalence using NDHS source.

Considering the bivariate model, which pools the two datasets, let *y*_*ij*_ be a binary indicator of HIV incidence at location *i* form dataset *j* = 1 , 2. Then *y*_*ij*_ ~ *Bernouilli*(*p*_*ij*_), *p*_*ij*_ is the probability of recorded HIV incident pertaining to the *j* ^*th*^ dataset. The vectors relating to all observations for the two responses were concatenated in

$$ Y=\left[\begin{array}{c}\hfill \begin{array}{cc}\hfill {y}_{11}\hfill & \hfill NA\hfill \\ {}\hfill \vdots \hfill & \hfill \vdots \hfill \\ {}\hfill {y}_{n_11}\hfill & \hfill NA\hfill \end{array}\hfill \\ {}\hfill \begin{array}{cc}\hfill NA\hfill & \hfill {y}_{12}\hfill \\ {}\hfill \vdots \hfill & \hfill \vdots \hfill \\ {}\hfill NA\hfill & \hfill {y}_{n_22}\hfill \end{array}\hfill \end{array}\right] $$, where *n*_*i*_ *j* is the number of observations for each response variable, *j* = 1 , 2.

Thus, the joint (bivariate) model is then given by;

5a $$ logit\left({p}_{i1}\right)={\beta}_{01}+{\sum}_k^r{\beta}_{k1}{x}_{i1 k}+{f}_1\left({g}_{i1}\right)+{z}_1\left({s}_i\right), $$

5b $$ logit\left({p}_{i2}\right)={\beta}_{02}+{\sum}_k^r{\beta}_{k2}{x}_{i2 k}+{f}_2\left({g}_{i2}\right)+{z}_2\left({s}_i\right)+\gamma {z}_1\left({s}_i\right), $$

where each response has a vector *x* of linear covariates with corresponding regression parameters *β*_*kj*_; *g*_*ij*_ is the vector of ages which are assumed to follow a random walk of order 1; *z*_1_(*s*_*i*_) is a Gaussian random field shared between both responses, the interaction parameter *γ* links the two response variables (i.e. HIV prevalence from NHSS and HIV prevalence from NDHS) and describes how much of the structure captured in *z*_1_(*s*_*i*_) is also inherent in the *logit*(*p*_*i*2_). Similar prior distributions to those specified for univariate models were assigned for parameters and hyperparameters of the joint model. A summary of models to be fitted in this study is provided in Table 2.

**Table 2:** Nested models to be fitted in this study

Model	Gaussian random field	Shared component	covariates
*M*_*U*1_: Univariate model for NDHS data	√	-	-
*M*_*U*2_: Univariate model for NHSS data	√	−	-
*M*_*U*12_: Univariate model for NDHS data + covariates	√		√
*M*_*U*22_: Univariate model for NHSS data + covariates	√		√
*M*_*J*1_: Bivariate model for NDHS and NHSS data	√	√	-
*M*_*J*2_: Bivariate model for NDHS and NHSS + covariates	√	√	√

### Estimation of parameters and model diagnostics

The estimation of parameters involved evaluation of the posterior distribution, which is the conditional distribution of the model parameters given the observed HIV data is obtained by taking the product of likelihood function together with the prior and hyper distributions. In this study, the posterior distribution is given by;

6 $$ p\left(\theta |{y}_{i j}\right)\propto {\Pi}_{i=1}^n L\left({y}_{i j},{p}_{i j}\right)\times {\Pi}_{g=1}^2\left[ p\left(\Delta {g}_i|{\tau}_g^{-1}\right) p\left({\tau}_g^{-1}\right)\right]{\Pi}_{k=1}^r p\left({\beta}_k\right) p\left({\tau}_{\beta}^{-1}\right){\Pi}_{j=1}^2 p\left({z}_j,{k}_j,{\nu}_j,{\sigma}_{z_k}\right) p\left(\gamma \right), $$

where *θ* is a vector of all parameters.

**Fig. 1:**
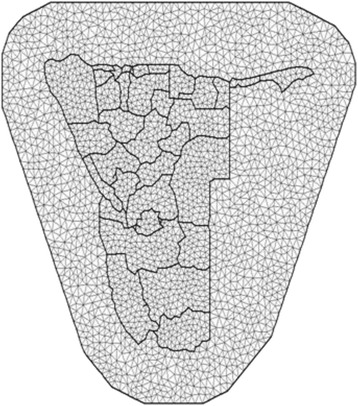
Convex hull meshes: Constrained refined Delaunay triangulation

A stochastic partial differential equation (SPDE) approach with R-INLA was employed to estimate posterior marginal distributions and any other posterior inferences. Convex hull meshes (Fig. 1) on study area were used in order to avoid the boundary effect [21]. Figure 1 presents the subdivision of the domain of study into a collection of non-intersecting triangles with condition that any two triangles meet at most a common edge or corner. The initial vertices are placed at the locations for observations and then additional vertices are added in a way that minimises the number of triangles needed to fill up the size and shape of the study domain of interest (Namibia). The polygon of triangles was extended out of the Namibia boundaries in order to avoid boundary effects. The best model was identified using the deviance information criterion (DIC) given by *DIC* = *D* + 2*p*, where *D* is the deviance evaluated at the posterior mean and *p* the effective number of parameters in the model. By the rule of thumb, the best model is one with the smallest value of DIC.

## Results

### Descriptive results

**Fig. 2:**
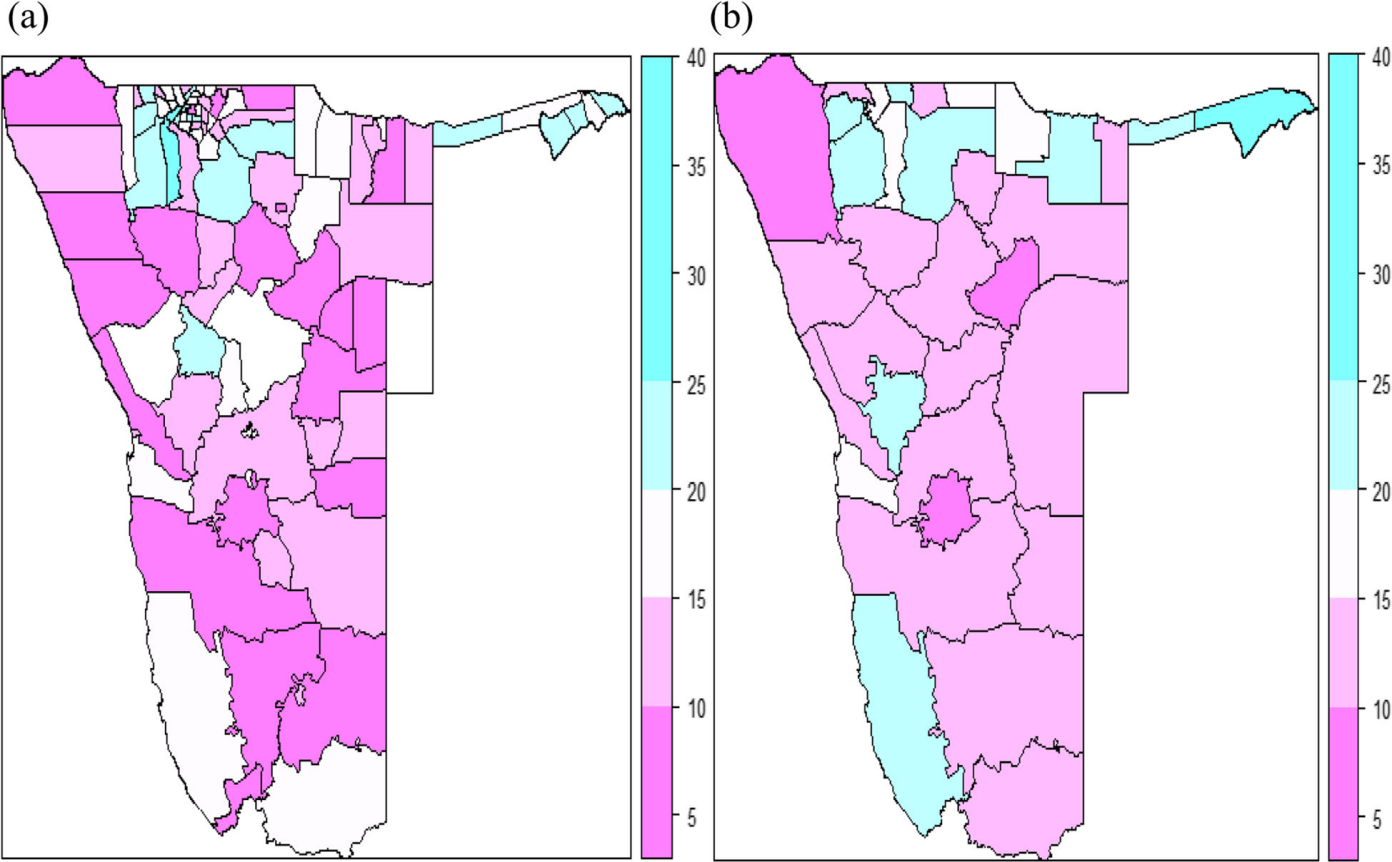
Observed HIV prevalence (in %): (**a**) constituencies’ HIV prevalence (2013 NDHS data); (**b**) health districts’ HIV prevalence (2014 NHSS data)

Figure 2(a) shows the spatial distribution of observed HIV prevalence in each constituency for women and men aged between 15 and 64 years obtained from the NDHS. This figure points out that there exist geographical (constituency level) differences of HIV prevalence in Namibia. Whereas Fig. 2(b) displays the geographical distribution of observed HIV prevalence among pregnant women aged 15–49 years attending antenatal care (ANC) clinics at public health facilities in Namibia (HIV Sentinel survey data). Two colours, namely purple and blue, were used to distinguish levels of HIV prevalence. The darker the purple, the lower is the observed HIV prevalence whereas the darker the blue, the higher is the HIV prevalence. From this figure, it can be noted that there exist spatial differences among health districts with respect to HIV prevalence. Summaries of HIV prevalence for both NDHS and NHS data sources are presented in Appendix 1 (Tables 5 and 6).

### Estimation of HIV prevalence

**Fig. 3:**
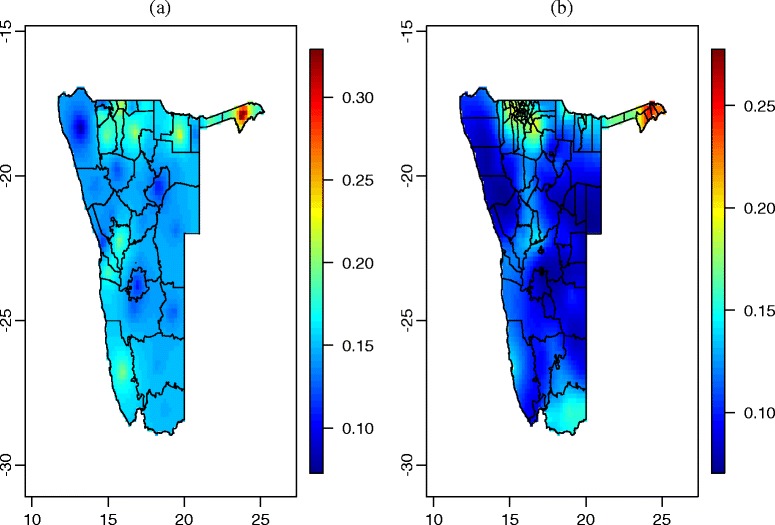
Estimated HIV prevalence using separate models: (**a**) HIV prevalence estimates from 2014 NHSS data; (**b**) HIV prevalence estimates from 2013 NDHS data

Figure 3(a) shows the estimated HIV prevalence within health districts using NHSS data source. From this figure, it can be deduced that in northern part of Namibia, Katima is estimated to have the highest HIV prevalence (30 to 35%). Furthermore, Andara, Rundu, Nakundu, Oshakati, Onandjokwe, okahao, Tsandi, Outapi, Eenhana, Kongo and Engela health districts, the HIV prevalence is estimated between 15 and 20%. In central west, Walvis Bay and Usakos health districts are estimated to be between 15 and 20% of HIV infection. In south, the HIV prevalence is estimated be around 15% in Ludertz. The rest of the health districts had reduced association with HIV infection (the prevalence is estimated be below 15%).

Figure 3(b) presents the estimates of prevalence derived from NDHS data using univariate model. High HIV infection is predicted to be associated with most of the constituencies in Caprivi region (25 to 30%). Other constituencies with elevated HIV prevalence are found in Omusati, Oshana, Oshikoto, and Kavango regions (15 and 20%). Karibib, Walvis Bay rural, Walvis Bay urban, and Luderitz were estimated to have approximately between 10 and 20% of HIV infection. The rest of constituencies are estimated to have HIV prevalence of around 10%.

**Fig. 4:**
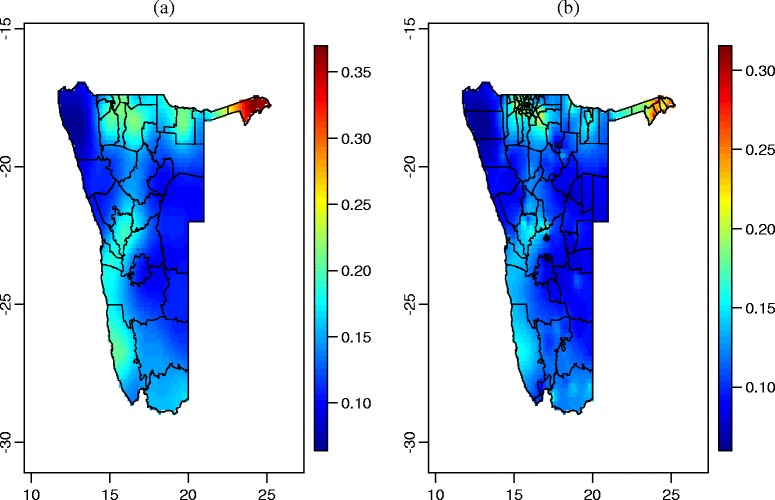
Estimated HIV prevalence using bivariate model: (**a**) HIV prevalence estimates from 2014 NHSS data; (**b**) HIV prevalence estimates from 2013 NDHS data

Figure 4 provides the estimates of HIV prevalence obtained from the bivariate model that pools the two datasets together. The bivariate model reveals an under estimation of HIV prevalence when NDHS source is used for estimation separated from NHSS source. Univariate model estimated the prevalence to be between 0 and 30%, whereas the bivariate model estimated the HIV prevalence to between 0 and 35%. For both data sources, the spatial distribution of HIV infection is very similar to the spatial distribution of HIV infection when univariate models were employed.

### Linear fixed effects and nonlinear effects

From Table 3, it can be noticed that model *M*_*j*2_ is the best model among all models. Thus, a summary statistics of this model is presented in Table 4 and the interpretation of the results is provided in the subsequent sections. The results of separate (univariate) model for each data set are provided in Appendix 1 (Tables 7 and 8), respectively.

**Table 3:** DIC values for fitted models

Model	DIC		
*M*_*U*1_: Univariate model for NDHS data (no covariate in the model)	7011.89		
*M*_*U*2_: Univariate model for NHSS data (no covariate in the model)	6872.59		
*M*_*U*12_: Univariate model for NDHS data + covariates	6344.00		
*M*_*U*22_: Univariate model for NHSS data + covariates	6388.33		
*M*_*J*1_: Bivariate model for DHS and NHSS data (no covariate in the model)	NDHS_DIC	NHSS_DIC	Total DIC
	7003.498	6870.218	13,873.716
*M*_*J*2_: Bivariate model for DHS and NHSS + covariates	NDHS_DIC	NHSS_DIC	Total DIC
	5998.112	6355.980	12,354.09

**Table 4:** Estimated covariate effects and their 95% credible intervals (CI)

Joint (bivariate) model		
Covariate	OR	95% CI
*β*_01_	0.12	(0.07, 0.23)
Prima-gravida(Ref)	1.00	
Multi-gravida	1.88	(1.52, 2.32)
*β*_02_	0.08	(0.04, 0.18)
Place of residence		
Rural(Ref)	1.00	
Urban	1.53	(1.27, 1.84)
Gender		
Female(Ref)	1.00	
Male	0.68	(0.58, 0.79)
Head of household		
Male (Ref)	1.00	
Female	1.14	(0.97, 1.33)
Martal status		
Never in union (Ref)	1.00	
Maried	0.72	(0.58, 0.89)
Living with a partner	1.41	(1.16, 1.73)
Widowed	1.46	(1.06, 2.02)
Divorced	1.07	(0.66, 1.75)
Separated	1.41	(1.04, 1.91)
Number of kids dead		
No child died (Ref)	1.00	
one child died	1.84	(1.48, 2.29)
More one than one child died	2.69	(1.84, 3.91)
Education		
No education (Ref)	1.00	
Primary	1.09	(0.87, 1.37)
Secondary	0.84	(0.66, 1.06)
Higher	0.63	(0.41, 0.96)
Wealth index		
Poorest (Ref)	1.00	
Poorer	0.93	(0.79, 1.09)
Middle	1.10	(0.89, 1.35)
Richer	0.78	(0.61, 0.99)
Richest	0.33	(0.24, 0.46)
Stayed away from home		
Did not move away (Ref)	1.00	
Moved away	0.93	(0.79, 1.09)
Sexual activity (in last 4 months)		
Never had sex (Ref)	1.00	
Not active	0.98	(0.90, 1.07)
Active	1.15	(1.06, 1.26)
Age at first sex		
Never had sex(Ref)	1.00	
< 11	1.29	(0.87, 1.91)
12–.14	1.08	(0.67, 1.73)
15–.17	1.47	(0.99, 2.17)
> 18 & at first union	1.26	(0.85, 1.87)
Condom used		
No (Ref)	1.00	
Yes	1.78	(1.53, 2.07)
Had STI in last 12 months		
No (Ref)		
Yes	1.05	(0.96, 1.16)

### HIV risk and its determinants: NHSS data

**Fig. 5:**
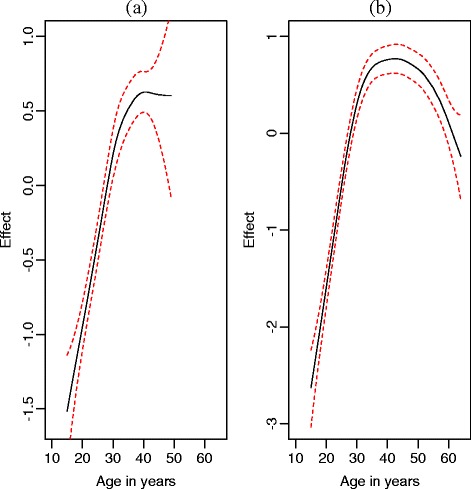
Estimated nonlinear effects of age on HIV infection and corresponding 95% credible intervals: (**a**) NHSS data; (**b**) NDHS data

For HSS data source, two covariates namely age and gravida were available at district level (Table 4). The age covariate was modelled using the first order random walk in order to deal with the nonlinearity whereas the gravida covariate was assumed to have linear effects on HIV. The odds of HIV infections among pregnant women with multi-gravida (mother had given birth to two more children) was 1.88 times as likely as that of women with prima-gravida (only one child born) (OR: 1.88, 95% CI: 1.52 to 2.32). Fig. 5(a) shows the relationship between the age of a pregnant woman and its effects on HIV infection. This figure showed that the likelihood of HIV infection follows a nonlinear growth trajectory (black lines indicates the nonlinear trajectory whereas the dotted lines represent its 95% credible interval). An increase in odds of HIV infection is observed up to a certain age and then it is followed by a decline in the risk of HIV infection.

### HIV risk and its determinants: NDHS data

For NDHS data, covariates on demographic, social, sexual behaviour, and biological characteristics were available and hence used in this study. Table 4 presents the results.

Place of residence classified as rural or urban was significantly related to HIV infection among men and women. The chance of HIV infection was lower for men and women residing in rural areas compared to those residing in urban areas (OR: 1.53, 95% CI: 1.27 to 1.84).

Gender was also found to be significantly associated with HIV infection. The likelihood of a man being infected was 0.68 times lower compared to that of a woman (95% CI: 0.58 to 0.79).

Head of a household was found to be significantly linked with HIV infection. Man or woman living in a household headed by a woman had higher risk of infection compared to one living in a household headed by a man (OR: 1.14, 95% CI: 0.97 to 1.33), though not significant.

Men and women who were married had less risk of infection compared to those who were never in union (OR: 0.72, 95% CI: 0.58 to 0.89). The likelihood for HIV was higher for widowers compered to men and women who were never in union (OR: 1.46, 95% CI: 1.06 to 2.02). The odds of HIV infection among men and women living with partners was 1.483 times higher than that of those who were never in union (OR:1.41, 95% CI: 1.16 to 1.73). Those who divorced had 1.07 times higher chance of infection relative to those who were never in union, though it is not significant (OR: 1.07, 95% CI: 0.66 to 1.75). The chance of HIV infection for those who separated or non-longer lived with their partners is 1.41 times higher than that of those who were never in union (OR:1.41, 95% CI: 1.04 to 1.91).

The likelihood of infection with HIV for men and women who had one of their children dead is as 1.84 times higher as those whom none of their children died (OR: 1.84, 95% CI: 1.48 to 2.29). Individuals who had more than one of their children dead were 2.69 times more likely infected with HIV relative to those who did not have any of their children dead (OR:2.69, 95% CI: 1.84 to 3.91).

Education was found to be negatively associated with HIV infection. The likelihood of testing positive was lower for men and women with secondary and or higher education as compared to those with no education. For instance, the odds of being infected with HIV was 0.63 times lower for men and women with higher education as compared to those with no education (OR:0.63, 95% CI: 0.41 to 0.96).

Wealth was found to be inversely associated with HIV infection. The chance of infection with HIV was 0.78 times less for those classified as richer than that of those classified as poorest (OR:0.78, 95% CI: 0.61 to 0.99). The men and women in the category of the richest had 0.33 times less likelihood of get HIV as compared to those in the category of the poorest (OR: 0.33, 95% CI: 0.24 to 0.46). Those in the middle class had 1.10 times odds of testing positive as compared to those in lowest class (OR: 1.10, 95% CI: 0.89 to 1.37), though not significant. Although not significant, individuals classified as poorer were 0.93 times less likely to test positive as compared to those classified as poorest (OR: 0.93, 95% CI: 0.79 to 1.09).

Table 4 also shows that sexual behaviour characteristics that include current sexual activity, condom use, and age at first sex were found to be related to HIV infection. Contrary to general myth, condom use was found to be positively related to HIV infections. Individuals who ever used condoms during their last sex with most recent partners were 1.78 times at higher risk of HIV infection as compared to those who did not use condoms during their last sex with most recent partners (OR: 1.78, 95% CI: 1.53 to 2.07).

Individuals with history of STI in the last 12 months were 1.05 times more likely to be HIV positive relative to those who did not contract STI in the last 12 months (95% CI: 0.96 to 1.16), though the difference is not significant.

People who had been away from their homes for more than one month in the last 12 months were found to be less likely to be HIV positive compared to those who did not go away from their homes for more than one month in last 12 months (OR: 0.93, 95% CI: 0.79 to 1.09), although the difference was also not significant.

Figure 5(b) shows that the odds of getting infected with HIV increases up to a certain age and then starts dropping at an increasing rate. This figure exhibits similar patterns to those shown in Fig. 5(a) except that the ages of respondents for Fig. 5(a) do not go beyond 49.

**Fig. 6:**
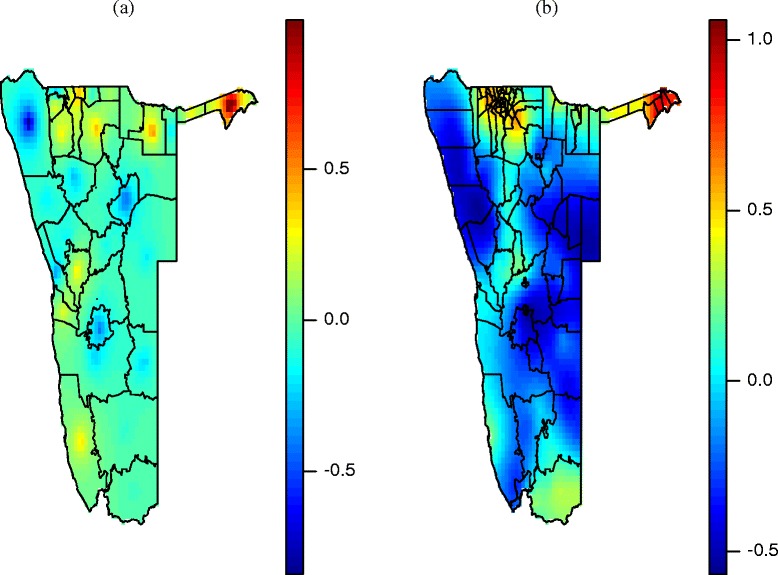
Posterior means of random effects from univariate models: (**a**) Spatial random effects (NHSS data); (**b**) spatial random effects (NDHS data)

**Fig. 7:**
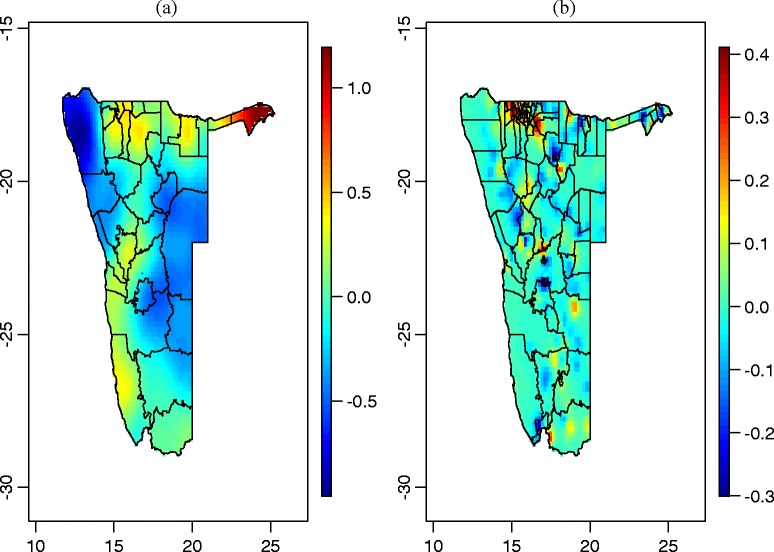
Posterior means of random effects from bivariate model: (**a**) Spatial random effects (NHSS data); (**b**) spatial random effects (NDHS data)

The maps of spatial random effects can be obtained from Figs. 6 and 7 in Appendix 2. Both figures show that health districts and constituencies in northern part of Namibia were more likely to be associated with HIV infection (i.e. positive posterior means of spatial random effects) whereas most of the rest of the health districts and constituencies had reduced association with HIV infection (i.e. negative posterior means spatial random effects).

## Discussion

In this study, a bivariate model controlling for spatial random effects was fitted. A full Bayesian framework through SPDE approach with INLA was implemented by jointly modelling the two data sources available at two different spatial levels. Thus, this joint model approach had to deal with data that were spatially misaligned. The bivariate model, which used a spatial shared component that acts as a surrogate of HIV risky behaviours among pregnant women in order to improve the estimation of HIV prevalence using NDHS source, was found to be more appropriate in estimating HIV prevalence. The interaction parameter *γ* = 2.14 (95% CI: 1.65 to 3.67), described how much of the structure captured in the shared component and also inherent in the NDHS HIV prevalence, was found to be significant. Hence, the joint analysis of NDHS and ANC sources has enhanced the estimation of HIV prevalence using the demographic and health survey (NDHS). This finding concurs with results from the study by Manda et al. [4].

As “everything that rises must converge” [22], it is argued that no quantity can grow for ever. Thus, the effect of age on HIV infection was considered to follow a growth trajectory with the two chronological patterns, namely a gradual increase from the beginning until the maximum is reached, and thereafter a gradual decrease. Consequently, it could have been inappropriate to assume that there is a linear relationship between age and the HIV infection. Therefore, in this study, the effect of age on HIV infection was modelled using semi-parametric regression.

For these two data sources, the relationship between age and its effects on HIV infection followed an inverted “U” shape. This finding agrees with other studies [6, 20].

The place of residence was found to be significantly associated with HIV infection. Individuals in urban areas had higher risk of getting infected compared those in rural areas. This finding has been reported in many other studies [4, 6, 20, 23]. It could be used to design focused public campaigns against HIV/AIDS such as campaigns for volunteer testing and use of condoms and antiretroviral based on the place residence.

This study had shown that poverty levels were inversely associated with the likelihood of HIV infection. People in middle class, rich class, and richest class had less risk of getting infected with HIV relative to those in lower class. In a similar study [24], unwanted or forced sex was related to lack of resources and the ability to obtain resources.

In this study, HIV infection was found to be significantly related with head of a household. Individuals living in a household headed by a woman were associated with higher risk of testing positive compared to the ones living in a household headed by a man. It has been shown that the male-headship is a proxy of a better socio-economic status [25], which had been proven to be inversely related to HIV infection. This finding could be explained by the complex of inferiority of women [26] and the struggle to obtain leadership positions and power to make decisions [24].

Another finding of this study is that gender was significantly associated with HIV infection. The likelihood of women to test HIV positive is high than that of men. Some of the possible explanations for this finding are gender inequality in the sex intimacy and relationship, multiple partners perceived as prestigious for boys, and complex of inferiority among girls in presence of boys [26]. The gender and HIV infection relationship was confirmed in many studies [4, 23, 24, 27].

It was found that the marital status impacts on the HIV infection. Widowers had high likelihood of being infected with HIV. One of the possible justifications for this finding could be that most widowers were left by partners who died of HIV. Though differences were not significant, odds of HIV infection were higher for divorced individuals and those who no-longer lived with partners compared to those who were never in union. This result could be useful in designing strategies and interventions intended to vulnerable groups especially widowers. Some earlier works had already indicated similar results [4, 6, 23, 27].

Another well-known finding in many studies [6, 20, 24], which was also found in this study is that education was negatively associated with HIV infection. The likelihood of testing positive was low among men and women with higher education as compared to those with no education. This could be due to fact that most of individuals with higher education are matured and aware of the danger of HIV and less sexually active. Though the difference is not significant, individuals with primary and secondary education were found be at high risk of contracting HIV as compared to those who never had any formal education. This finding could be related to limited sexual education in Namibia schools. Although life-skills programs tailored to equip learners with knowledge about sexuality education are implemented in Namibia schools, it has been argued that there is no proper training provided to teachers in this matter and also that students do not take this subject seriously as it is not examinable [26]. As Namibia government is committed to provide education to all Namibia [28], this finding could be used by the Government to realise the need of extending free education to other phases of formal education in order to increase the number of potential individuals who will eventually achieve high education. Also, it could be used as an indicator of a need to revise the life- skills curriculum and implementation of exams for this subject in order to encourage learners to take it seriously.

Contrary to general myth, condom use was found to be positively related to HIV infections. Individuals who ever used condom during the last sex with most recent partner were at higher risk of contracting HIV as compared to those who did not condom. This unexpected result was also reported in the work of Ngesa, Mwambi and Achia [20]. One of the justifications provided to this finding was that men use condoms in the earlier stage of relationship with their partners and later on give up on using condoms. Another possible justification to this finding could be that many of the condom users knew their HIV status (positive) and make use of condoms to protect their partners.

It was also found that the number of kids dead had a positive significant effect on HIV infection. The likelihood of getting infected with HIV for men and women who had one or more of their children dead was higher than that of those whom none of their children died. This might imply that kids could have been infected by their mothers. With respect to this outcome, the Ministry of Health and Social Services should redouble its efforts in the implementation of prevention of mother-to-child transmission of HIV/AIDS programmes until the mother to child transmission rate which was about 2% in 2013 [29] drops to 0%.

With respect to sexual behaviour or biological characteristics such as sexual activity, age at first sex and STI, this study has found that these characteristics of sexual or biological behaviour are associated with HIV infection. This result could be used to identify groups with high risk where greater efforts should be directed.

In disease mapping, the identification of areas correlated with high risk proves to be useful in designing preventive and intervention strategies such as HIV testing campaign, accessibility and use of condoms, antiretroviral treatment, and efficient budget allocation. According to the findings of this study, great efforts in terms of primary and secondary HIV interventions should be concentrated to constituencies in the northern part of Namibia.

This study had made use of shared component through the SPDE approach to analyse jointly the two sources of data and it presented two major strengths. Firstly, the joint modelling approach developed in this study allowed to combine two data sources that are available at different spatial levels in a single model. Secondly, unlike other studies that assumed a same underlying spatial process for different sources, with the bivariate model developed it is possible to specify different spatial processes (e.g. a Poisson and Bernoulli processes) through the link function.

A number of significant weaknesses of this study are acknowledged. Firstly, due to confidentiality issues, the positions of HIV cases were random displaced in the NDHS data source. This study did not take into account the bias that might be induced by such displacements. Therefore, the interpretation of the study findings should take into account this limitation. Secondly, the missingness is quite common in NDHS and NHSS data sets. This might somehow distort the geographical distribution pattern of disease. Nevertheless, we hope that the spatial smoothing approach employed in this study might have lessened an aberrant.

## Conclusion

This study had shown determinants of HIV infection in Namibia and had revealed areas at high risk of HIV infection through HIV prevalence mapping. The findings from this study and the prevalence maps produced could be used by the Ministry of Health and Social Services and any health policy makers to identify groups of people in need of HIV support and where they live in order to efficiently allocate resources that are increasingly becoming scarce. Moreover, the study had used a bivariate modelling approach that helped dealing with spatially misaligned data. Additionally, the study has shown that the prediction of HIV prevalence using the DHS data source can be enhanced by jointly modelling other HIV data such as NHSS data.

## Appendix 1

**Table 5:** Observed HIV prevalence per constituency and per gender

Region	Constituency	% Prevalence (Men)	% prevalence (Women)	Combined % Prevalence
Caprivi	Kabe	12.90	35.29	24.62
	Katima Mulilo Rural	7.50	23.81	15.85
	Katima Mulilo Urban	22.86	40.24	32.24
	Kongola	5.26	24.00	15.91
	Linyanti	22.22	25.00	23.81
	Sibinda	12.00	32.00	22.00
Erongo	Arandis	6.67	7.69	7.14
	Daures	0.00	0.00	0.00
	Karibib	12.50	15.15	13.85
	Omaruru	17.78	27.27	21.79
	Swakopmund	14.17	14.29	14.23
	Walvis Bay Rural	10.91	20.83	16.54
	Walvis Bay Urban	9.72	7.79	8.72
Hardap	Gibeon	4.17	12.00	8.16
	Mariental Rural	12.50	9.68	10.91
	Mariental Urban	9.09	12.90	10.94
	Rehoboth East Urban	7.50	6.12	6.74
	Rehoboth Rural	12.50	5.56	8.82
	Rehoboth West Urban	0.00	4.55	2.27
Karas	Berseba	0.00	16.67	9.38
	Karasburg	12.50	19.05	16.22
	Keetmanshoop Rural	0.00	6.25	3.23
	Keetmanshoop Urban	12.00	14.89	13.40
	Luderitz	16.67	15.63	16.07
	Oranjemund	3.57	3.85	3.70
Kavango	Kahenge	14.29	16.44	15.65
	Kapako	13.16	8.16	10.34
	Mashare	0.00	8.33	3.70
	Mpungu	6.25	22.50	15.28
	Mukwe	22.50	18.00	20.00
	Ndiyona	4.00	15.79	11.11
	Rundu Rural East	9.38	25.49	19.28
	Rundu Rural West	10.71	17.46	14.29
	Rundu Urban	31.58	25.00	27.66
Khomas	Katutura Central	7.27	13.41	10.95
	Katutura East	0	0	0
	Khomasdal North	4.11	3.7	3.91
	Moses ||Garoeb	23.3	20.86	22.32
	Samora Machel	2.84	10.78	7.64
	Soweto	0	20.75	8.94
	Tobias Hainyeko	24.02	25	24.43
	Windhoek East	0	0	0
	Windhoek Rural	13.04	13.04	13.04
	Windhoek West	0	2.13	1.34
Kunene	Epupa	0	16.67	7.69
	Kamanjab	9.52	5.88	7.89
	Khorixas	4.76	6.25	5.66
	Opuwo	10	12	11.11
	Outjo	17.86	11.43	14.29
	Sesfontein	15.38	0	7.69
Ohangwena	Eenhana	6.9	7.89	7.46
	Endola	14.29	22.5	19.67
	Engela	10.2	34.25	24.59
	Epembe	30	10	18
	Ohangwena	16.67	15.79	16.22
	Okongo	2.86	12.5	8.79
	Omulonga	7.14	20	14.13
	Omundaungilo	0	28.57	14.29
	Ondobe	2.78	24.19	16.33
	Ongenga	8.33	16.22	13.11
	Oshikango	4.08	25.97	17.46
Omaheke	Aminuis	5.26	5.56	5.41
	Epukiro	8.33	0	5.26
	Gobabis	10.53	10	10.26
	Kalahari	20	4.76	11.11
	Otjinene	5.88	4.17	4.88
	Otjombinde	0	0	0
	Steinhausen	7.14	10	8.33
Omusati	Anamulenge	11.76	16.67	14.63
	Elim	25	27.78	26.47
	Etayi	14.89	15.28	15.13
	Ogongo	9.09	20.51	16.39
	Okahao	18.75	20.83	20
	Okalongo	16.67	21.43	19.81
	Onesi	8	14.29	11.32
	Oshikuku	0	35.29	15.79
	Otamanzi	20	29.63	26.19
	Outapi	17.39	22.67	20.66
	Ruacana	18.18	12.12	15.58
	Tsandi	9.09	30.3	20.66
Oshana	Okaku	8.7	22.86	17.24
	Okatana	7.69	36.36	20.83
	Ompundja	16.67	58.33	44.44
	Ondangwa	11.67	28.09	21.48
	Ongwediva	8.24	10.42	9.39
	Oshakati East	18.18	22.86	20.8
	Oshakati West	16.67	18.18	17.58
	Uukwiyu	4.17	21.43	13.46
	Uuvudhiya	11.76		11.76
Oshikoto	Eengondi	17.65	28.21	22.22
	Guinas	11.11	7.14	10.17
	Okankolo	5.26	22.22	10.71
	Olukonda	26.67	4.55	13.51
	Omuntele	9.09	26.47	19.64
	Omuthiyagwiipundi	19.57	27.08	23.4
	Onayena	10.53	24.32	19.64
	Oniipa	17.5	8.77	12.37
	Onyaanya	6.25	14.29	10.81
	Tsumeb	1.85	6.78	4.42
Otjozondjupa	Grootfontein	12.77	17.24	15.24
	Okahandja	12.82	17.24	15.46
	Okakarara	5.71	7.41	6.45
	Omatako	0	5.56	2.56
	Otavi	7.14	13.04	9.8
	Otjiwarongo	13.19	13.48	13.33
	Tsumkwe	12.5	12.5	12.5

**Table 6:** Observed HIV prevalence per health district/site

Heath district/site	Tested	Negative	Positive	Prevalence
Andara	255	204	51	20.00
Aranos	138	122	16	11.59
Eenhana	215	187	28	13.02
Engela	259	200	59	22.78
Gobabis	158	138	20	12.66
Grootfontein	222	191	31	13.96
Karasburg	214	183	31	14.49
Katima Mulilo	375	240	135	36.00
Keetmanshoop	163	140	23	14.11
Khorixas	180	157	23	12.78
Luderitz	278	220	58	20.86
Mariental	191	168	23	12.04
Nankudu	195	164	31	15.90
Nyangana	279	244	35	12.54
Okahandja	195	169	26	13.33
Okahao	228	181	47	20.61
Okakarara	156	142	14	8.97
Okongo	263	217	46	17.49
Omaruru	170	148	22	12.94
Onandjokwe	299	232	67	22.41
Opuwo	155	149	6	3.87
Oshakati	286	234	52	18.18
Oshikuku	306	249	57	18.63
Otjiwarongo	236	202	34	14.41
Outapi	254	225	29	11.42
Outjo	188	167	21	11.17
Rehoboth	154	140	14	9.09
Rundu	303	230	73	24.09
Swakopmund	210	188	22	10.48
Tsandi	277	221	56	20.22
Tsumeb	257	219	38	14.79
Usakos	119	93	26	21.85
Walvisbay	219	176	43	19.63
Windhoek	330	284	46	13.94
Overall	7727	6424	1303	16.86

**Table 7:** Fixed effects and their 95% credible intervals (CI): Separate model for NDHS data

Covariate	OR	95% CI
*β*_02_	0.08	(0.03,0.21)
Place of residence		
Rural(Ref)	1.00	
Urban	1.57	(1.30, 1.89)
Gender		
Female(Ref)	1.00	
Male	0.67	(0.58, 0.78)
Head of household		
Male (Ref)	1.00	
Female	1.14	(0.97, 1.33)
Marital status		
Never in union (Ref)	1.00	
Maried	0.72	(0.58, 0.89)
Living with a partner	1.43	(1.17, 1.75)
Widowed	1.49	(1.07, 2.05)
Divorced	1.09	(0.67, 1.76)
Separated	1.44	(1.06, 1.95)
Number of kids dead		
No child died (Ref)	1.00	
one child died	1.86	(1.49, 2.31)
More than one child died	2.74	(1.88, 3.99)
Education		
No education (Ref)	1.00	
Primary	1.09	(0.87, 1.38)
Secondary	0.85	(0.67, 1.08)
Higher	0.63	(0.41, 0.96)
Wealth index		
Poorest (Ref)	1.00	
Poorer	1.10	(0.89, 1.36)
Middle	1.00	(0.80, 1.25)
Richer	0.77	(0.60, 1.00)
Richest	0.32	(0.23, 0.46)
Stayed away of home		
Did not move away (Ref)	1.00	
Moved away	0.93	(0.79, 1.08)
Never had sex (Ref)	1.00	
Not active	0.98	(0.90, 1.07)
Active	1.15	(1.06, 1.26)
Age at first sex		
Never had sex(Ref)	1.00	
<11	1.29	(0.89, 1.96)
12–.14	1.09	(0.68, 1.76)
15–.17	1.49	(1.01, 2.23)
>18 & at first union	1.28	(0.87, 1.92)
Condom used		
No (Ref)	1.00	
Yes	1.78	(1.53, 2.07)
Had STI in last 12 months		
No (Ref)		
Yes	1.06	(0.96, 1.16)

**Table 8:** Fixed effects and their 95% credible intervals (CI): Separate model for NHSS data

Covariate	OR	95% CI
*β*_01_	0.12	(0.09,0.17)
Prima-gravida(Ref)	1.00	
Multi-gravida	1.89	(1.52,2.34)

## Abbreviations

AIDS: Acquired Immunodeficiency Syndrome
ANC: Antenatal Clinic
CI: Credible Interval
NDHS: Namibia Demographic and Health Survey
DIC: Deviance Information Criterion
EA: Enumeration area
HIV: Human Immunodeficiency Virus
IGLS: Iterative Generalised Least Squares
INLA: Integrated Nested Laplace Approximation
NHSS: National HIV Sentinel Surveillance
MACR: Multivariate Conditional Autoregressive
MoHSS: Ministry of Health and Social Services
OR: Odds Ratio
SPDE: Stochastic Partial differential Equation
STI: Sexually Transmitted Infection
UNAIDS: Joint United Nations Programme on HIV/AIDS
WHO: World Health Organization
